# Estimation Accuracy of Root Canal Curvatures from Different Dental Diagnostic Imaging Techniques: An In Vitro Experimental Study

**DOI:** 10.1155/2021/6699635

**Published:** 2021-01-13

**Authors:** Bestoon Mohammed Faraj

**Affiliations:** Conservative Department, College of Dentistry, University of Sulaimani, Madame Mitterand Street 30, Sulaimani, 46001 Kurdistan Region, Iraq

## Abstract

In clinical endodontics, preoperative estimation of root canal curvature is crucial regarding the prevention of iatrogenic errors. Reproduction of the two-dimensional radiographic images causes certain proximal view curvatures not seen. Therefore, the present study is aimed at investigating the degree of root canal curvature identified in different radiographic views. A total of 60 human permanent single-rooted teeth with varying degrees of curvature were selected. The root canal curvature for each tooth was measured on cone-beam computed tomography (CBCT) images (clinical view), standard digital periapical view (0° angle), digital periapical horizontal parallax view (30° angle), and digital periapical proximal view (0° angle), by using the Schneider method. No statistically significant difference was found in the degree of curvatures estimated on CBCT images and standard digital periapical view (0° angle) in the same tooth. The results revealed a significant difference between the proximal view and the other three groups (*p* < 0.05). There was no significant difference in this respect between the horizontal parallax view (30° angle), clinical view (CBCT images), and standard digital periapical view (*p* > 0.05). Proximal view curvatures cannot be predicted or estimated only from examining a clinical view radiograph. A horizontal parallax view (30° angle) is highly recommended as specific guidelines on how to estimate root canal curvature in case difficulty assessment protocols.

## 1. Introduction

In clinical endodontics, diagnostic high-quality radiographic imaging is essential for the assessment of root canal anatomy. Based on a radiograph, the restorability of a tooth and the complexity of the root canal morphology, and necessary treatment protocols can be evaluated [1]. The morphology of the curved root canal is crucial for the shaping outcome of root canal instrumentation and chemomechanical preparation, and it is directly related to the clinical success of root canal treatment. When the degree of root canal curvature's angle increases, the screwing action of the rotary file becomes greater, the risk of breakage increases, and the possibility of iatrogenic errors also increases. The canal curvature's angle exceeding 30° lead to complications in root canal preparation and subsequent obturation of radicular spaces [2]. The preoperative estimation of the canal length in multirooted teeth becomes more complicated as the degree of root curvature increases [3, 4].

In restorative dental practice, the complex intricacy of the root canal system is hard to comprehend and thus not feasible for dental practitioners. The level of case difficulty increases with increasing degrees of root curvature [5]. Precise information about the shape and degree of curvature of root canals directs the mind of a clinician to give attention to apply a suitable treatment protocol specific to instrument selection and instrumentation technique and helps to prevent possible problems. This information is necessary not only in a mesial to distal direction, as seen in standard buccolingual (clinical) view radiograph, but also in a buccal to the lingual direction (proximal view radiograph). Although in clinical endodontics, canal curvature in the proximal view is unseen clearly by the clinician with standard intraoral parallel radiographic technique; it can play a significant role in the clearly defined treatment outcomes. Many researchers reported that curves in the mesiodistal plane often are greater than those in the more readily accessible buccolingual plane [6, 7].

Several experimental studies have shown a significant discrepancy between what was found anatomically and seen radiographically [8]. The parallax technique is an obvious variation or difference in the apparent position or location of an object caused by the change of position on a two-dimensional or three-dimensional point of observation [9, 10].

Cone-beam computed tomography (CBCT) systems, too, are a practical device for noninvasive and three-dimensional reconstruction of radiographic imaging for uses in the endodontic examination and diagnosis application and as a research tool in morphologic analyses. Cone-beam computed tomography is a 3-dimensional imaging system that overcomes the limitations of conventional and digital periapical radiographs [11, 12].

In endodontic practice and in laboratory investigations, several methods are available to measure root canal curvature's angle depending on imaging factors, though there is an obvious lack of a consensus on the ideal technique to achieve this goal [13]. It is reasonable to expect that few studies have focused on the evaluation of the root canal curvature's angle viewed from localization techniques. Also, no specific guidelines are available on how to estimate the degree and angulation of root canal curvature in most commonly used case difficulty assessment forms. This *ex vivo* study is aimed at comparing and evaluating the impact of different radiographic images on the assessment of root canal curvature, CBCT image (clinical view), standard digital periapical view (0° angle), digital periapical horizontal parallax view (30°), and digital periapical proximal view (0° angle) and providing an appraisal for their clinical applications.

## 2. Materials and Methods

### 2.1. Ethical Approval and Experimental Design

The protocol of this study was approved by the ethics institutional committee at Sulaimani University, before the initiation of the laboratory procedures (approval no. 415/2020). This study has followed the CRIS guidelines (Checklist for Reporting in vitro Studies), as discussed in the 2014 concept note [14]. A master list was made for only 60 specimens out of 85 extracted teeth previously collected for this study from different public hospitals and private clinics within Sulaimani and Hawler Cities, between December 2019 and June 2020. The following categories were used as inclusion criteria: fully formed single-rooted tooth with varying degrees of curvature. A preliminary examination for determination of root curvature direction and angulation for all the teeth have been performed by visual inspection over a transparent grid, whether the root was straight, curved towards mesial, distal, buccal, lingual, or angulated in multiple directions. A tooth with uncommon extreme variations like twisted buccal root or three fused roots was excluded. In this manner, the other 25 teeth did not fulfill the inclusion criteria. An endodontist performed the selection and preparation of the samples. There was no information about the patients' age, gender, tooth quadrant, or reason for extraction. The specimens are cleaned and stored in 10% formalin for a maximum of 2 months. Finally, they were kept in normal saline before use.

### 2.2. Digital Intraoral Radiographic Techniques

All periapical digital and CBCT images were obtained by an experienced oral and maxillofacial radiologist calibrated to the standard criteria of tracing and measurements. The teeth were embedded in the radiolucent polysiloxane impression putty dental impression material (3 M ESPE, St. Paul, MN, USA), and this is to ensure that each specimen kept in a steady position and adjusted concerning the three simulated absolute positions (buccolingual direction (clinical view), horizontal parallax 30° angle, and mesiodistal direction (proximal view)). The long axis of the root was mounted parallel and as close as possible to the surface of the X-ray sensor (EzSensor Classic, Vatech, Korea). Periapical radiographs were obtained for all the teeth using a standardized parallel technique with the aid of a film holder (XCP; Rinn, Elgin, IL). A high-frequency oral X-ray machine (EzRay Air W; Vatech, Korea) was used with an exposure time of 0.367 seconds (60 kV, 4 mA). To compensate for image magnification, the target–receptor distance was increased to ensure that only the most parallel rays are directed toward the tooth and the X-ray sensor. As a result, a long (16-inch) target–receptor distance was used [15]. The images were collected and analyzed using an open-source image analysis program (EasyDent V4 Viewer, Vatech, Korea), by a single calibrated operator.

### 2.3. Canal Curvature Measurements on CBCT Radiographic Images

Every ten teeth were arranged consistently inside a custom-made wood box with the aid of a dental plaster (3 M ESPE, USA). The placement of the specimens in the specially constructed box during imaging ensured that all images were standardized and reproducible. The specimens were scanned from the buccolingual section of each tooth by using CBCT (NewTom Giano, Verona, Italy) with 90 kVp, 3 mA, voxel size (0.3 mm), and FOV 8 cm by 11 cm. Each mold was horizontally aligned to the chin support in a way that the occlusal plane adjusted into a correct parallel position to the plate holder [16].

**Figure 1:**
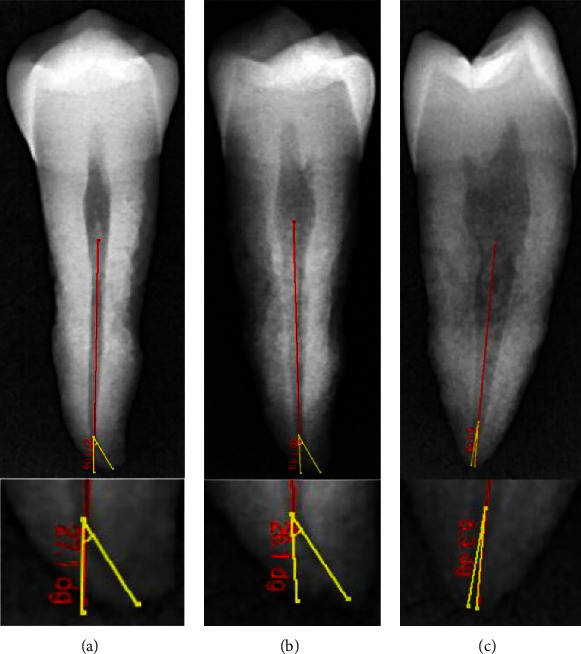
Representative digital radiographic images demonstrating a canal curvature angle measurement for a tooth without root angulation using a Schneider method. (a) 0° angle buccolingual view. (b) 30° angle horizontal parallax view. (c) 0° angle proximal view.

**Figure 2:**
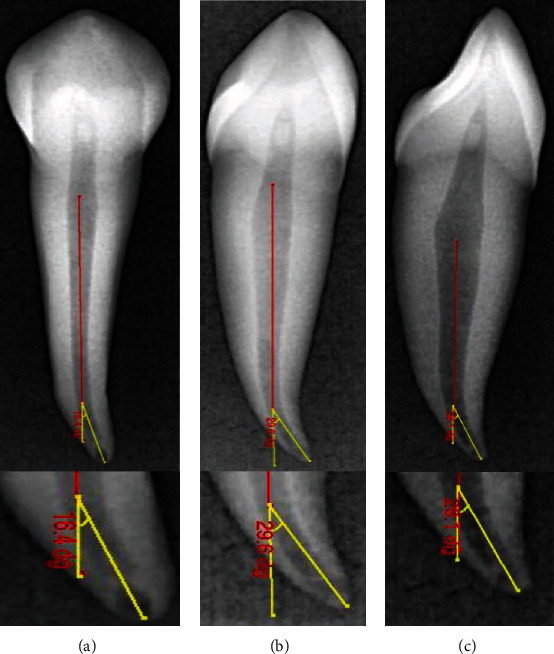
Representative digital radiographic images demonstrating a canal curvature angle measurement for a tooth with root angulation using a Schneider method. (a) 0° angle buccolingual view. (b) 30° angle horizontal parallax view. (c) 0° angle proximal view.

**Figure 3:**
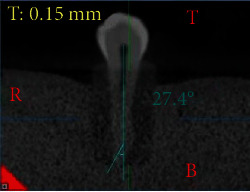
Representative CBCT radiographic image demonstrating a canal curvature angle measurement for a tooth without root angulation using a Schneider method.

The degree of root canal curvature's angle was measured on all the radiographic images captured for the same 60 teeth scanned (Figures 1–3). For the CBCT evaluation, scan images from the clear sagittal view were selected depending on the multiplanar imaging-reformatted sections. The slices were first reproduced in a vertical position to visualize the tooth cusp, pulp chamber, apical foramen, and the complete view of the root canal pathway. All images are converted for viewing with the image analysis software (NNT Software, Verona, Italy) to measure the canal curvature angle.

### 2.4. Canal Curvature Measurement on Digital Radiographic Images

The degree of root canal curvature's angle was determined by using the Schneider technique, which measures curvature as the acute angle between the long axis of the root canal and a line joining the apical foramen to the point of the initial canal curvature. The Schneider method involves first drawing a line parallel to the long axis of the canal, in the coronal third; a second line is then drawn from the apical foramen to intersect the point where the first line left the long axis of the canal. The Schneider angle is the intersection of these lines [7].

### 2.5. Statistical Analysis

The collected data were analyzed with SPSS version 18.0 (SPSS Inc., Chicago, IL). The sample size was determined with the Sealed Envelope software for a power of 80% and the level of significance of approximately 5%. The normal distribution of the data was tested using the Shapiro-Wilk test.

The means and standard deviations (SD) were calculated for each group. One-way analysis of variance and Tukey's post hoc tests were used for multiple comparisons. A *p* value of less than .05 was considered statistically significant. The Pearson correlation coefficient was calculated based on the data from the CBCT scans and digital radiography to evaluate the association between root angulation and degree of curvatures. A *p* value of less than .05 was considered statistically significant.

## 3. Results

The degree of root canal curvature determined by Schneider's method for all the 60 teeth examined from different radiographic views on digital and CBCT images are summarized in Table 1 and Figures 1–3. The recorded means of the degree of root canal curvature of all specimens from the highest to the lowest value were as follows: 21.73° with a clinical view (CBCT), 20.65° with horizontal parallax view, 19.37° with a clinical view (digital), and 6.36° with a proximal view.

**Figure 4:**
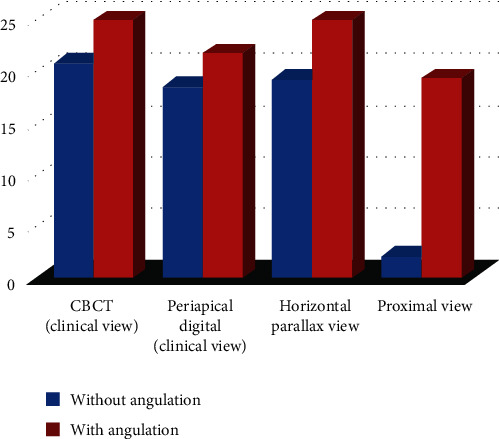
The bar chart shows the mean value (canal curvature angle) of each group with and without buccal or lingual angulation.

Overall, in clinical view images, only 19 (6.35%) of the samples exhibited moderate or severe curvature, but in proximal view, 52 (17.39%) of the samples had an angle of canal curvature larger than 15 degrees. The maximum mean value of the angle of canal curvature recorded was 21.73 degrees in clinical view (CBCT), and the minimum mean value was 6.36 degrees with proximal view radiograph (Table 1 and Figure 4).

The one-way analysis of variance (ANOVA) and post hoc tests demonstrated a significant difference between the proximal view and the other three groups (*p* < 0.05). There was no significant difference in this respect between 30° angle horizontal parallax and clinical views (CBCT and digital) (*p* > 0.05) as shown in Table 2.

**Figure 5:**
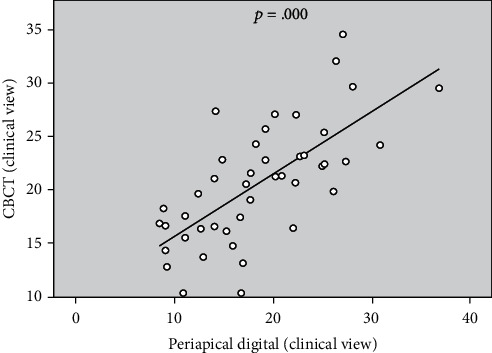
The significant positive correlation of degree of canal curvature measured on CBCT and digital periapical radiographic images for specimens with buccal or lingual angulation.

**Figure 6:**
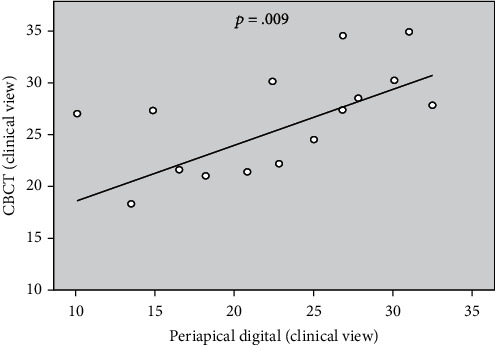
The significant positive correlation of degree of canal curvature measured on CBCT and digital periapical radiographic images for specimens without buccal or lingual angulation.

The calculations were performed for the teeth with buccal or lingual angulation (*n* = 15), and a significant correlation (*p* > 0.15) was observed between CBCT and digital clinical views (Table 3 and Figure 5). For the teeth without buccal or lingual angulation (*n* = 45). A statistically significant correlation (*p* > 0.15) was also noted between CBCT and digital clinical views (Table 4 and Figure 6). The correlation demonstrated the direct relationship in the degree of curvature between the CBCT and digital periapical radiograph when viewed from a clinical radiograph. Pearson correlation coefficients were then calculated for the horizontal parallax compared with the proximal view. However, the degree of canal curvature correlation coefficient was not statistically significant at the 0.05 level.

## 4. Discussion

In clinical endodontics, the degree of root canal curvature's angle is closely related to endodontic case difficulty assessment. Despite the availability of three-dimensional imaging techniques, the mainstream preoperative estimation of root canal configuration is depending mostly on the evaluation of single or multiple high-quality periapical radiographs [17]. These images allow visualization of curvatures in the mesiodistal direction, but will not fully disclose features of the root canal system in the buccolingual direction, thus masking root canal complexities which cause root canal treatment to be less predictable [18, 19]. This is a common limitation of various techniques proposed in the literature, despite the lack of detailed guidelines for root canal curvature classification in widely used case difficulty assessment form (American Association of Endodontists 2019) [20]. Alteration of beam angulation might provide additional information not readily available from the buccolingual view image [21, 22]. And this is to overcome the anatomic variations that may be found in each tooth [23, 24].

This study investigated the estimation accuracy of different X-ray views for identifying the degree of root canal curvature angle of single-rooted canine and premolar teeth, by using a CBCT and digital periapical radiograph as a research tool. When compared to two-dimensional imaging, CBCT presents a greater accuracy regarding the determination of root canal morphology [19, 25, 26]. In this study, canal curvature measurement on CBCT (clinical view) images revealed a significantly more accuracy than periapical digital images (clinical view) and proximal view, for all the examined teeth. Neither of the buccolingual and mesiodistal root angulations, CBCT showed a significant difference with the digital periapical horizontal parallax view (30°).

To obtain precise and consistent results, only one calibrated operator carried out the tracing and measurements on the CBCT and digital images. The intraoral radiographs were captured using a digital system. This produced a dynamic image allowing the user to alter brightness and contrast easily. The buccolingual projection (clinical view) was included in the present study because it is widely used as a standard protocol in the estimation of root canal morphology in clinical practice.

Magnification can be minimized by keeping the object as close to the film as possible, but a certain amount of magnification is unavoidable. Distortion can be minimized by using a parallel technique to assist in positioning the object in the central part of the X-ray beam; however, properly angled radiographs provide a buccolingual view with higher diagnostic accuracy in determining the number of roots and canals [27].

Pearson correlation coefficients were calculated in different radiographic views, to determine whether the root curvature directions (buccal or lingual angulation) correlated with its degree of curvature. The analysis indicated that the measurement on the clinical view and horizontal parallax view exhibited the best diagnostic accuracy in identifying the highest degree of canal curvature and the second-highest accuracy for identifying a degree of root canal curvature in teeth with buccal or lingual root angulations. However, the proximal view is clinically impossible and revealed the highest degree of curvature for root with buccal angulation.

The result of this study made it possible to find out much more information than from the 2-dimensional clinical view or proximal view. Clinicians might be able to recognize clearly the 3-dimensional root canal system's anatomy, choose the right treatment plan, and properly interpret prognosis. The degree of curvature throughout the sample set varied widely, with great differences between the maximum and minimum values of angle. This indicates that a radiographic view might contribute to a clinician's underestimation of the degree of difficulty of endodontic therapy. Proximal view curvatures cannot be predicted or estimated only from examining a clinical view radiograph. Many curved canals might be mistaken for straight ones, or canals with secondary curvature might be mistaken for canals with a single curvature, causing the clinician to underestimate the difficulty of the root canal preparation.

This study drew attention to the estimation accuracy of the degree of canal curvature with clinical view radiograph and 30° horizontal parallax radiograph and the possible correlation between anatomical classification of root angulations and radiographic view. Furthermore, highlighted the most effective horizontal angulation for successful estimation of the degree of curvature of the root canal in single-rooted teeth. These basic findings are consistent with research showing that careful evaluation of two or more periapical radiographs is mandatory. These angled radiographs provide additional detail about root canal morphology not obtained by other projections [28–30]. However, our findings differ from the study of Naoum et al. [21], who suggested that 0-degree radiographs provided more information than 30-degree radiographs. The discrepancy might result from differences in the study objectives and methods.

The present study found that 30-degree mesial radiographs were significantly better than 0° angle clinical view radiographs for visibility and estimation of the optimum degree of proximal view curvature. Further research must be conducted to compare the radiographic views in multirooted teeth and more severely curved canals. These findings might provide clues for clinicians to understanding the radiographic view for accurate estimation of the degree of curvature in case difficulty assessment form before the initiation of the endodontic procedure.

The limitations of the present studies need to be considered, such as the selection of a single root canal, and the extreme variation in root morphology was not taken into account as independent factors on analysis. We did not find any research articles similar to our study methodology about preoperative estimation accuracy of root canal curvature angle. However, we acknowledge that there are considerable discussions among researchers to develop a protocol involving optimum angles for horizontal angulations for inspection of the actual degree of root canal angle and direction of root angulation. We suggest conducting this study with an advanced technique such as a micro-CT system and in vivo study with more samples.

## 5. Conclusions

Despite the limitations of this study, this experiment adds to a growing literature that the 30° angle horizontal parallax method remains an accurate method for the estimation of the degree and direction of root canal angulations. These findings would enable the operator a more predictable and accurate estimation of the degree of root canal curvature and assign a level of difficulty of a particular case before the endodontic procedure.

## Figures and Tables

**Table 1:** The mean and standard deviation (SD) values of the degrees of the root canal curvature angles measured on radiographic images reproduced by different techniques.

Descriptive statistics					
Radiographic view	*N*	Minimum	Maximum	Mean	Std. deviation
Clinical view (CBCT)	60	10.10	34.90	21.73	5.84701
Clinical view (digital)	60	8.50	36.90	19.37	6.82056
Horizontal parallax view	60	10.20	41.30	20.65	7.12592
Proximal view	60	.00	45.00	6.36	10.18612

**Table 2:** Multiple comparisons between the measurements obtained on digital periapical and CBCT radiographic images for all tested specimens.

Groups	Compared with	Mean difference (I-J)	Std. error	*p* value^∗^
Clinical view (CBCT)	Clinical view (digital)	2.35833	1.40013	.334
	Horizontal parallax view	1.07667	1.40013	.868
	Proximal view	15.36833^∗^	1.40013	.000

Clinical view (digital)	Horizontal parallax view	-1.28167	1.40013	.797
	Proximal view	13.01000^∗^	1.40013	.000

Horizontal parallax view	Proximal view	14.29167^∗^	1.40013	.000

^∗^One-way analysis of variance.

**Table 3:** Pearson correlations between the radiographic views for the estimation of the degree of root canal curvature angle for the teeth with buccal or lingual angulation.

		Clinical view (CBCT)	Clinical view (digital)	Horizontal parallax view	Proximal view
Clinical view (CBCT)	Pearson correlation		.647^∗∗^	.333	.380
	(*p* value) sig. (2-tailed)		.009	.226	.163
	*N*	15	15	15	15

Clinical view (digital)	Pearson correlation	.647^∗∗^		.232	.251
	(*p* value) sig. (2-tailed)	.009		.406	.367
	*N*	15	15	15	15

Horizontal parallax view	Pearson correlation	.333	.232		.429
	(*p* value) sig. (2-tailed)	.226	.406		.110
	*N*	15	15	15	15

Proximal view	Pearson correlation	.380	.251	.429	
	(*p* value) sig. (2-tailed)	.163	.367	.110	
	*N*	15	15	15	15

^∗∗^Correlation is significant at the 0.05 level (2-tailed).

**Table 4:** Pearson correlations between the radiographic views for the estimation of the degree of canal curvature angle for the teeth without buccal or lingual angulation.

		Clinical view (CBCT)	Clinical view (digital)	Horizontal parallax view	Proximal view
Clinical view (CBCT)	Pearson correlation		.692^∗∗^	.289	-.066
	(*p* value) sig. (2-tailed)		.000	.054	.665
	*N*	45	45	45	45

Clinical view (digital)	Pearson correlation	.692^∗∗^		.275	.058
	(*p* value) sig. (2-tailed)	.000		.067	.705
	*N*	45	45	45	45

Horizontal parallax view	Pearson correlation	.289	.275		-.023
	(*p* value) sig. (2-tailed)	.054	.067		.881
	*N*	45	45	45	45

Proximal view	Pearson correlation	-.066	.058	-.023	
	(*p* value) sig. (2-tailed)	.665	.705	.881	
	*N*	45	45	45	45

^∗∗^Correlation is significant at the 0.05 level (2-tailed).

## Data Availability

The numerical data used to support the findings of this study are available from the corresponding author upon request.
